# A cyamella causing popliteal tendonitis

**DOI:** 10.1308/003588414X13824511649931

**Published:** 2014-01

**Authors:** N Rehmatullah, R McNair, J Sanchez-Ballester

**Affiliations:** St Helens and Knowsley Teaching Hospitals NHS Trust,UK

**Keywords:** Cyamella, Popliteal, Sesamoid, Tendonitis, Meniscal

## Abstract

A 64-year-old man presented with intermittent but progressive lateral-sided knee pain. Symptoms mimicked those of a lateral meniscal tear. Magnetic resonance imaging revealed a cyamella associated with popliteal tendonitis and an intact lateral meniscus.

## Case history

A 64-year-old male maintenance manager presented to the accident and emergency (A&E) department with a 4-month history of intermittent pain in the posterolateral aspect of his right knee after a simple twisting injury. The pain worsened as the day progressed and the knee swelled over the following 24 hours but resolved without treatment.

For the few months prior to this incident, the patient experienced several episodes of pain followed by giving way but no locking. He did not report any snapping. He had a complicated past medical history of gout, atrial fibrillation (on warfarin), diffuse B-cell lymphoma, colonic carcinoma and stage III renal disease. Examination by an A&E consultant revealed a moderate sized effusion and lateral joint line tenderness. The range of movement was 0–100º and the McMurray test was positive. All other joints were normal. Radiography of the knee showed no abnormalities and routine haematological, biochemical and immunological tests were normal (international normalised ratio: 1.9). A diagnosis of acute-on-chronic osteoarthritis was made and a corticosteroid injection was given.

The patient returned to the emergency department two weeks later with increasing pain. Physical examination, blood tests and repeat x-ray were identical. Aspiration of joint fluid was negative for organisms, pus and crystals. An orthopaedic knee consultant reviewed the patient and a diagnosis of lateral meniscus tear was made. Outpatient magnetic resonance imaging (MRI) was ordered and he was discharged home with analgesia.

**Figure 1:**
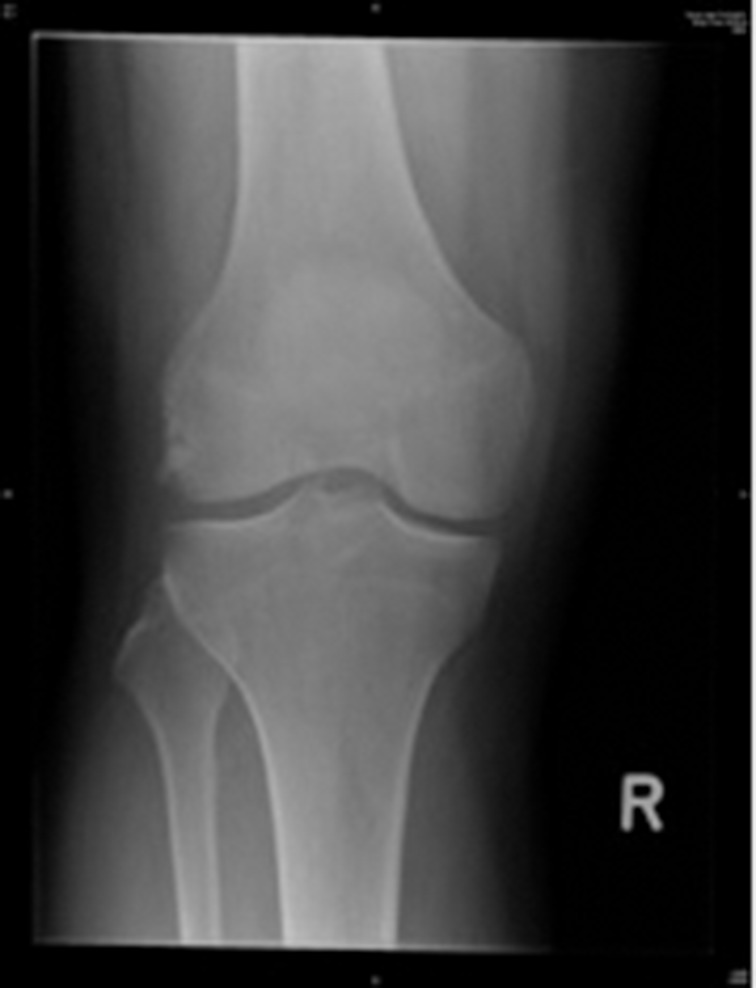

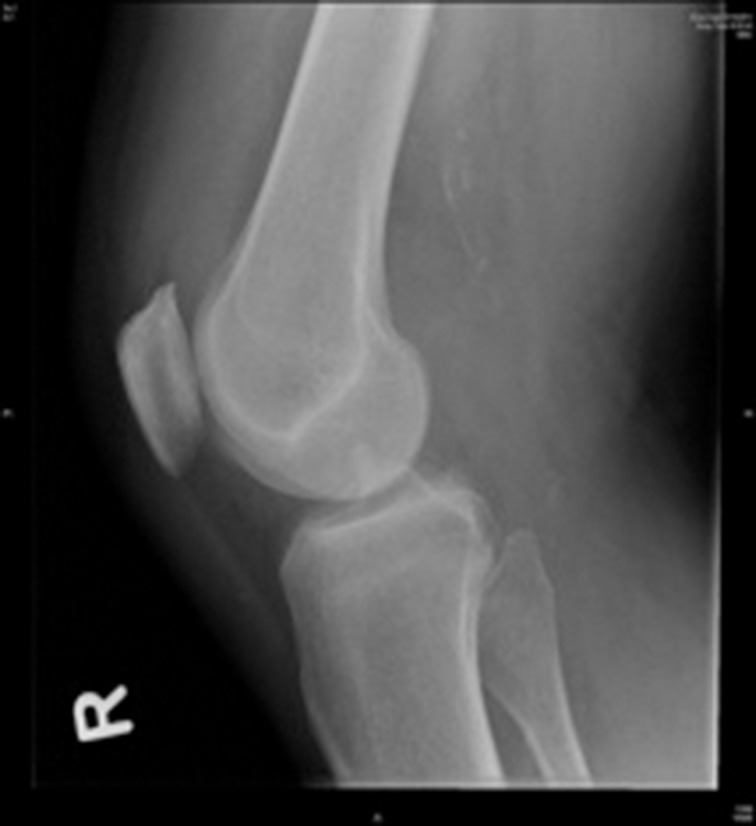
Anteroposterior and lateral knee x-ray taken at initial presentation, demonstrating cyamella behind lateral femoral condyle

Four weeks later, another orthopaedic consultant reviewed the patient. On examination, varus stress testing was positive but the McMurray test was negative and all other findings were identical. An x-ray showed a 2cm × 1cm well corticated abnormality on the lateral aspect of the lateral femoral condyle (Fig 1) and a diagnosis of partial avulsion of the lateral collateral ligament was made. The patient was injected with 10ml of 1% lignocaine at the site of injury, with minimal benefit.

**Figure 2:**
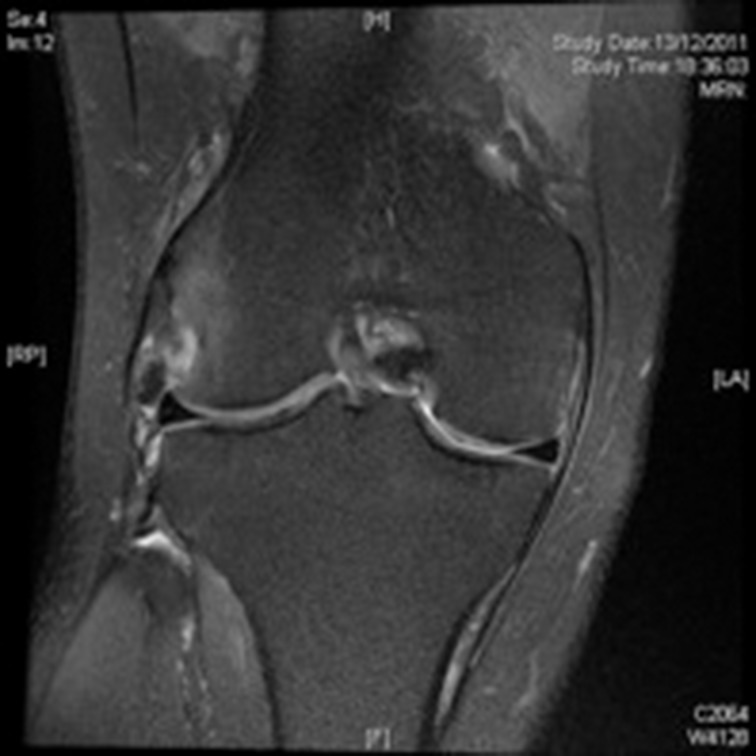

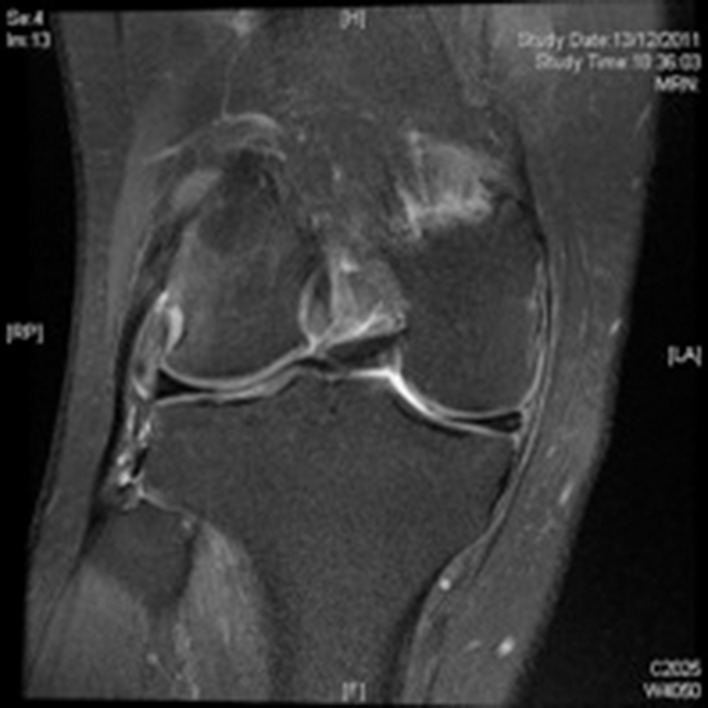
Cyamella causing popliteal tendonitis with surrounding oedema, three weeks following initial presentation

The MRI (Fig 2) reported the presence of a cyamella at the musculotendinous junction of the popliteus tendon with significant oedema surrounding the tendon and in the lateral femoral condyle. The menisci were normal. A diagnosis of impingement of the popliteus tendon secondary to the cyamella with resultant popliteal tendonitis was made. The patient was placed in a hinge brace for six weeks to prevent popliteal irritation. This provided substantial pain relief. Symptoms reoccurred (although to a lesser degree) once the brace was removed. A decision not to excise the cyamella was made due to the patient’s significant medical co-morbidities.

## Discussion

Sesamoid bones are small accessory bones residing in tendons and muscles. Their action is usually to facilitate the physiological motion of the tendon by increasing the excursion distance. However, they are also known to cause considerable pathology.

One such example is a cyamella, which is located in the popliteus tendon (below the joint line) or at the musculotendinous junction (above the joint line).^1^ It is seen commonly in primates and dogs but very rarely in humans. When it does occur in humans, it can articulate with the lateral condyle of the tibia and lies very close to the head of the fibula.^2^ This should not be confused with a fabella, which lies in the lateral head of the gastrocnemius.^3^

Very few cases of symptomatic cyamellas have been reported to date. Dheer *et al* described locking in a 14-year-old boy secondary to a cyamella^1^ while Mishra and Jurist reported a symptomatic dislocated cyamella,^4^ and Benthien and Brunner described a recreational athlete with posterolateral knee pain and restricted movement, in the absence of trauma.^5^ Krause and Stuart reported a 21-year-old woman with a similar clinical presentation but this was secondary to a snapping popliteal tendon.^6^ The snapping occurred consistently at 20–30º of knee flexion and was caused by the tendon rubbing over a prominent tubercle of the popliteal sulcus. In this case, surgical excision of the tubercle resolved symptoms.

In our case, the patient’s symptoms are likely to have been initiated by a simple traumatic episode resulting in symptoms mimicking a lateral meniscal tear. The MRI findings of oedema in the lateral femoral condyle and in the tendon surrounding the cyamella indicate irritation of the popliteal tendon with the lateral femoral condyle secondary to the cyamella. It is therefore possible that this was a case of popliteal tendonitis secondary to a cyamella. Unlike other reports where recurrent dislocation of the cyamella is hypothesised,^5^ we believe this was not the case in our patient. Recurrent dislocation would be associated with recurrent acute symptomatology, which he did not have. However, we cannot exclude that a dislocation of the cyamella occurred at the time of the initial injury.

## Conclusions

Orthopaedic surgeons and general practitioners who encounter patients with persistent undiagnosed lateral knee pain should bear in mind the diagnosis of a cyamella.
